# Alterations in frontal white matter neurochemistry and microstructure in schizophrenia: implications for neuroinflammation

**DOI:** 10.1038/tp.2015.43

**Published:** 2015-04-14

**Authors:** J Chiappelli, L E Hong, S A Wijtenburg, X Du, F Gaston, P Kochunov, L M Rowland

**Affiliations:** 1 Department of Psychiatry, Maryland Psychiatric Research Center, University of Maryland School of Medicine, Baltimore, MD, USA

## Abstract

We investigated *in vivo* neurochemical markers reflective of neuronal health and glial activation to determine if these could yield clues regarding the reduced fractional anisotropy (FA) of white matter and accelerated decline of FA with age in schizophrenia. Participants with schizophrenia and healthy controls completed diffusion tensor imaging to assess FA and proton magnetic resonance spectroscopy to assess neurochemical metabolites in the same frontal region. Frontal FA was significantly lower in the schizophrenia and declined more rapidly with age compared with the healthy control group. In both groups, *N*-acetylaspartate (NAA), a putative marker of neuronal integrity, and glutamate declined with age, and this decline was stronger in patients. Myo-inositol, a marker of glial cells, was negatively related to FA in both groups. The relationship between FA and age remained significant in schizophrenia even when controlling for all metabolites. The relationships of FA, NAA and myo-inositol to age appear to be independent of one another. The relationship between FA and myo-inositol was independently present in both patients and controls, even after controlling for age, indicating a potential general effect of neuroinflammation on white matter microstructure. Further studies are warranted to determine the underlying mechanism driving the accelerated FA decline with age in schizophrenia.

## Introduction

Growing evidence suggests white matter dysfunction is part of the pathophysiology in schizophrenia.^1, 2, 3^ Compromised white matter function likely contributes to the synchronization and connectivity abnormalities observed in schizophrenia.^4^ Fractional anisotropy (FA) of water diffusion, measured using diffusion tensor imaging (DTI), reflects the ‘integrity' of the white matter and is a replicable imaging finding in schizophrenia.^5, 6, 7, 8, 9^ The lifetime FA trajectory parallels developmental myelination changes and may index the normal aging process of the white matter.^3, 4, 10, 11, 12, 13^ Some data indicate that schizophrenia patients have stronger age-related decline in FA compared with controls, suggesting an accelerated white matter neurodegenerative process in schizophrenia;^3^ this finding is not consistent, however, with some studies indicating FA decline in patients becomes similar to that of controls with advanced age.^14, 15^ The biological mechanisms of reduced FA and greater decline with age in schizophrenia are unclear.

Using proton magnetic resonance spectroscopy (^1^H-MRS), we previously demonstrated that white matter *N*-acetylaspartate (NAA) concentrations were capable of explaining age-related FA variances in normal controls.^16^ In this study, we used ^1^H-MRS to examine NAA plus additional metabolites from the frontal white matter of schizophrenia patients and age-matched healthy controls. The largest signals detected by this technique include NAA, choline (Cho) and creatine (Cr). NAA is synthesized in neuronal mitochondria and highly concentrated in neuronal bodies and axons.^17^ NAA is believed to be a marker of neuronal function, may act as an osmolyte^18^ and may have a role in myelin synthesis through donation of its acetate group.^19^ Cho is made up of glycerophosphocholine, a cellular membrane metabolite, and phosphocholine, a cellular membrane precursor.^20^ In healthy individuals, Cho concentration in frontal white matter remains stable with aging.^16, 21, 22, 23^ The Cr signal, made up of creatine and phosphocreatine, is commonly observed to be stable over time.^16, 24^ Therefore, this project focused on white matter NAA but not Cho or Cr.

We also examined other metabolites known to be associated with neuroinflammation and aging: myo-inositol and glutamate. Myo-inositol is highly concentrated in astrocytes;^25^ it is an essential precursor for the phosphatidylinositol cell signaling pathway as well as for synthesis of membrane phospholipids.^26^ Increased myo-inositol is associated with glial activation and macrophage infiltration during chronic inflammation and slow virus infections of the brain.^27, 28^ In prototypical white matter autoimmune disorders such as multiple sclerosis, increased myo-inositol is observed in almost all studies even in normal appearing white matter.^29, 30^ Increased white matter myo-inositol has been found in an animal model of hypomyelination,^31^ and many other types of white matter^32, 33, 34^ and neuroinflammatory disorders.^35^ Myo-inositol increases with normal aging,^36^ thought to be due to white matter demyelination and associated glial proliferation. Previous studies in patients with schizophrenia have shown no changes in myo-inositol in frontal white matter in younger patients compared with age-matched controls,^37, 38^ but mixed results in older patients, with one study finding increased myo-inositol^37^ and another finding decreased myo-inositol compared with controls.^22^ However, the possible relationship of white matter myo-inositol to the accelerated, age-related decline of FA in schizophrenia has not been reported.

Elevated cortical glutamate has been observed in early/acute psychosis and unmedicated states but decreases in aging/chronic schizophrenia.^39^ White matter MRS of glutamate showed that acute psychosis^40^ and schizophrenia in elderly patients^22^ were both associated with higher glutamate levels. However, the aging trend of glutamate level in the white matter of schizophrenia patients has not been reported.

The aim of this study was to examine the underlying neurochemical factors that may explain the schizophrenia-related frontal FA deficits and the accelerated FA decline with age, with particular focus on whether putative neuroinflammatory biomarkers such as myo-inositol explain some of the white matter FA anomalies in schizophrenia. FA offers insight into the changes in the micro-structural integrity of white matter tracts.^41, 42^ Its decline in aging and psychiatric/neurological disorders is associated with an increase in the water diffusion across the myelin sheath and this is interpreted as evidence for dysmyelination/demyelination, reduction in the density of oligodendrocytes and/or replacement of axonal fibers with glial cells, all of which are potential consequences of neuroinflammation.^43, 44, 45, 46^ We examined metabolites and FA in the left frontal anterior corona radiata (ACR). ACR is an associative cortico-cortical frontal white matter tract and relays higher-order cognitive information.^47^ ACR's FA values experience precipitous decline in normal aging,^48, 49, 50^ possibly because the oligodendrocytes that myelinate this tract have reduced rates (per axonal-segment) of myelin production and repair^51^ and are highly susceptible to metabolic damage.^52^ Using the left ACR as the region of interest (ROI), we tested the hypothesis that white matter metabolites may provide a biochemical mechanism to explain the reduced FA and the faster age-related FA decline in schizophrenia. This study sought to answer two questions: (1) Do metabolites measured using ^1^H-MRS, especially those associated with neuroinflammation, contribute to reduced FA in schizophrenia? (2) Do these metabolites measured using ^1^H-MRS contribute to the accelerated age-related decline in FA in schizophrenia patients?

## Materials and methods

### Participants

Patients were recruited from the outpatient clinics at the Maryland Psychiatric Research Center and the neighboring mental health clinics. Healthy controls were recruited through media advertisements. Diagnoses were confirmed with the Structured Clinical Interview for DSM-IV in all the participants. Major medical and neurological illnesses, history of head injury with cognitive sequelae and mental retardation were exclusionary. All participants were interviewed using Structured Clinical Interview for DSM-IV substance use diagnoses. Patients and controls with substance dependence within the past 6 months or current substance abuse (except nicotine) were excluded. Except three medication-free participants, all schizophrenia patients were on antipsychotic medications. Controls had no current DSM-IV Axis I diagnoses and no family history of psychosis in the prior two generations. Participants gave written informed consent and this study was approved by the University of Maryland Institutional Review Board.

This study included DTI, ^1^H-MRS and clinical and neuropsychological testing in 38 persons with schizophrenia (age range 20–58 years) and 36 age- and sex-matched community controls (age range 20–61 years). Clinical data were available in all the participants. The DTI data from one control and one patient and MRS data from one control and seven patients were deemed of insufficient quality due to excessive motion or local lesion and were removed before data analysis. Psychiatric symptoms were assessed with the 20-item Brief Psychiatric Rating Scale total score,^53^ and cognitive capacities were assessed by processing speed (digit symbol coding subtest of the WAIS-3)^54^ and working memory (digit sequencing task).^55^ Processing speed is considered one of the most robust cognitive domain deficits in schizophrenia.^56, 57^ The demographics, cognitive, functional and symptom assessments of the samples are shown in Table 1.

### Diffusion tensor imaging

All imaging was performed at the University of Maryland Center for Brain Imaging Research using a Siemens 3T TRIO MRI (Erlangen, Germany) system equipped with a 32-channel phase array head coil. The high-angular resolution diffusion imaging DTI data were collected using a single-shot, echo-planar, single refocusing spin-echo, T2-weighted sequence with a spatial resolution of 1.7 × 1.7 × 3.0 mm. The sequence parameters were: TE/TR=87/8000 ms, FOV=200 mm, axial slice orientation with 50 slices and no gaps, five b=0 images and 64 isotropically distributed diffusion weighted directions with *b*=700 s mm^−2^. These parameters maximized the contrast to noise ratio for FA measurements.^58^ A tract-based spatial statistics method, distributed as a part of FMRIB Software Library package, was used for tract-based analysis of diffusion anisotropy.^44^ First, FA images were created by fitting the diffusion tensor to the motion and eddy current diffusion data. RMSDIFF^59^ was used to estimate the root mean square movement distance between diffusion sensitized and *b*=0 images. All the data passed QA control of <3 mm accumulated motion during the scan. There was no difference in the average motion per TR between patients and controls (0.42±0.21 vs 0.43±0.20, for patients and controls, respectively). In the next step, all FA images were globally spatially normalized to the Johns Hopkins University^60^ and then nonlinearly aligned to a group-wise, minimal-deformation target brain using the FLIRT method.^44, 61^ The group's minimal-deformation target brain was identified by warping all individual brain images in the group to each.^62^ Next, individual FA images were averaged to produce a group-average anisotropy image. This image was used to create a group-wise skeleton of white matter tracts. The skeletonization procedure was a morphological operation, which extracts the medial axis of an object. Finally, FA images were thresholded at FA=0.20 level to eliminate non-white matter voxels, and FA values were projected onto the group-wise skeleton of white matter structures. This step accounts for residual misalignment among individual white matter tracts. FA values were assigned to each point along a skeleton using the peak value found within a designated range perpendicular to the skeleton. This processing was performed under two constraints. A distance map was used to establish search borders for individual tracts. The borders were created by equally dividing the distance between two nearby tracts. Second, a multiplicative 20 mm full width at half-maximum Gaussian weighting was applied during the search to limit maximum projection distance from the skeleton. The average FA from the left ACR was used as the primary FA measure, using the same voxel used for MRS acquisition (see below). Whole-brain averaged FA was also explored.

### White matter MRS

**Figure 1:**
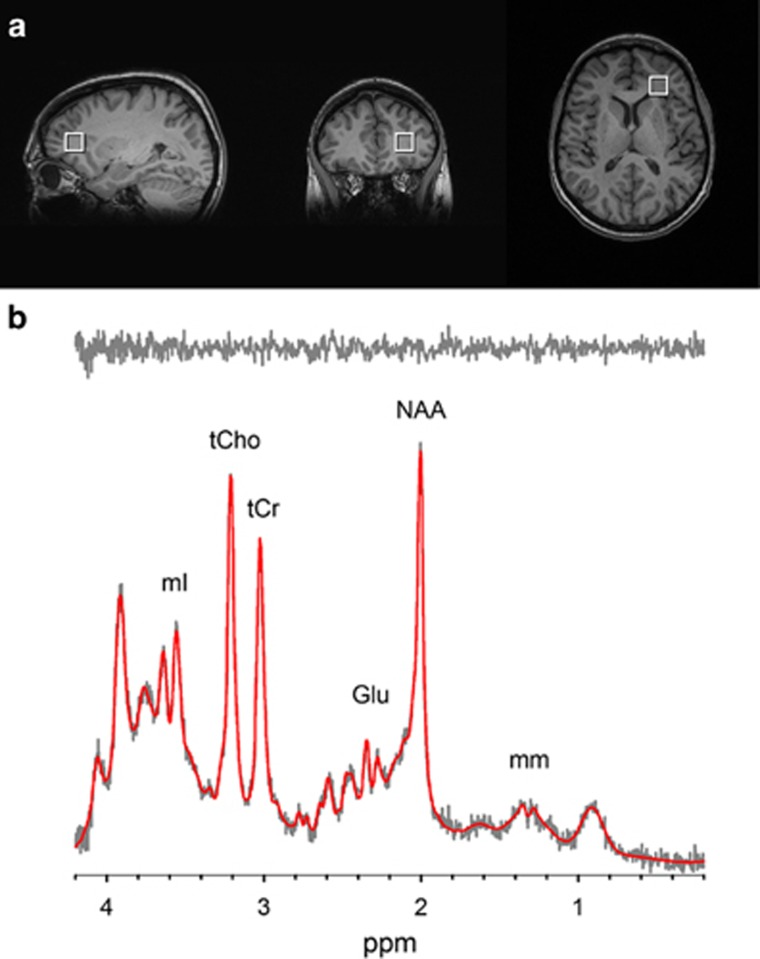
(**a**) T1-weighted images showing voxel placement in frontal white matter. (**b**) Representative spectrum (gray) and corresponding LCModel fit (red) from the frontal white matter voxel. The fit residual (shown above in gray) reveals that all metabolites and macromolecules have been accounted for, suggesting an excellent fit. Glu, glutamate; mI, myo-Inositol; NAA, *N*-acetylaspartate; tCho, glycerophosphocholine plus phosophocholine; tCr, creatine plus phosphocreatine.

A spectroscopic voxel was placed in the white matter of the forceps minor area of the left hemisphere (that is, left ACR) to avoid cerebrospinal fluid and gray matter (Figure 1a). The same voxel was used to calculate the left frontal white matter averaged FA. Single-voxel PRESS localization was utilized with the following parameters: TR/TE=2000/30 ms, VOI~3.4 cm^3^, NEX=256, 2048 complex points, 1.2 kHz spectral width and total scan time~12 min. A water reference (NEX=8) was collected and utilized for phasing and eddy current correction. A basis set was simulated using the GAVA software package,^63^ which was modified to yield a Lorentzian lineshape instead of its default Gaussian lineshape. The basis set was made up of alanine (Ala), aspartate (Asp), Cr, γ-aminobutyric acid (GABA), glucose (Glc), glutamine (Gln), glutamate (Glu), glycine (Gly), glycerophosphocholine (GPC), glutathione (GSH), lactate (Lac), myo-Inositol (mI), NAA, *N*-acetylaspartylglutamate (NAAG), phosphocholine (PCh), phosphocreatine (PCr), phosphoroylethanolamine (PE), scyllo-Inositol (sI) and taurine (Tau). This basis set was imported into LCModel, a fully automated curve fitting software package^64^ for metabolite quantification (Figure 1b). Metabolites were corrected for the proportion of the gray matter, white matter and cerebrospinal fluid within the spectroscopic voxel using in-house Matlab code.^65^ All metabolite concentrations were relative to the water reference and are reported in institutional units. The exclusion criteria for this data were: (1) signal-to-noise ratio reported by LCModel was ⩽10; (2) full width at half-maximum reported by LCModel was ⩾0.09; and (3) metabolite fits with Cramer Rao Lower Bounds (CRLBs) >20%. All spectra were of good quality with a mean full width at half-maximum of 0.044 for controls and 0.049 for patients and a mean SNR of 28 for controls and 26.3 for patients. The metabolites consistently identified with CRLBs <20% and used in statistical analyses were: NAA plus NAAG (NAA; mean CRLBs=2.7 (control), 3.1 (patient)), Cr plus phosphocreatine (Cr; mean CRLBs=2.9 (control), 3.1 (patient)), glycerophosphocholine plus phosophocholine (Cho; mean CRLBs=4.2 (control), 4.3 (patient)), myo-inositol (mean CRLBs=5.3 (control), 5.1 (patient)), and glutamate (mean CRLBs=7.3 (control), 7.4 (patient)).

### Data analysis

Group comparisons for clinical and imaging measures were performed using one-way analysis of variance. Group categorical comparisons used two-sided *χ*^2^ tests. Distributions of all the imaging data did not deviate from normal distribution by Kolmogorov–Smirnov tests, so correlation analyses were performed using Pearson's correlations. Partial correlations were performed to test whether age contributed to the relationship between FA and white matter metabolites. After the bivariate investigations, imaging variables shown to be related to age and/or schizophrenia were entered into an omnibus linear multiple regression to examine the relative contributions of age, diagnosis and any of the ACR white matter metabolites on the FA variations. All quantitative predictors in the model were centered by their respective means before they were entered into the model.^66^ Collinearity was assessed by the variance inflation factors. A variance inflation factor >4 usually indicates potential problem of collinearity.^67^ A Fisher *r*-to-*z* transformation of the correlation coefficients was used to compare the strength of the correlations between groups. Finally, we repeated the above linear multiple regression using whole-brain FA. All the tests were two-tailed with significance set to *P*<0.05 and Bonferroni corrected when appropriate.

## Results

### Clinical information and patient–control differences

Schizophrenia patients showed significantly reduced FA in the left frontal ACR ROI (*P*=0.015) compared with normal controls. There were no significant differences between the groups in concentration of metabolites as measured with MRS (Table 1).

### Relationship with age

**Figure 2:**
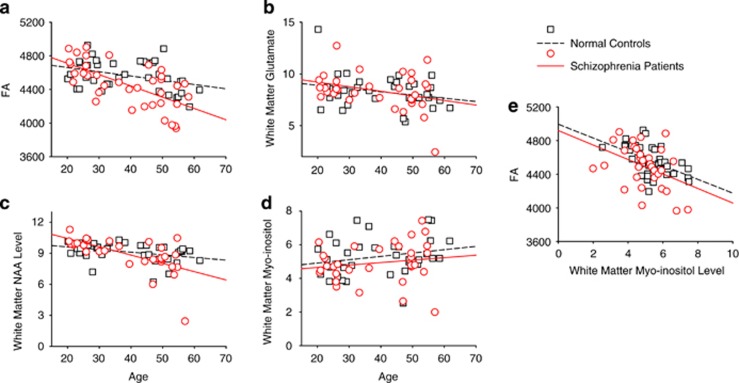
(**a**–**d**) Age effect on frontal white matter FA (**a**), white matter glutamate (**b**), white matter NAA (**c**) and white matter myo-inositol (**d**). Relationship between frontal white matter FA and white matter myo-inositol (both are from the left anterior corona radiata) are highlighted in **e**. FA, fractional anisotropy; NAA, *N*-acetylaspartate.

Age was significantly associated with whole-brain average FA, frontal FA and three metabolites, glutamate, myo-inositol and NAA measured in the frontal ROI in the combined sample (data not shown) and independently in normal controls and schizophrenia patients (Table 2 and Figures 2a and d).

### Relationship of FA and white matter metabolites

In the combined group, whole-brain and ACR FA were significantly and positively correlated with frontal white matter tNAA (*r*=0.414, *P*<0.001 and *r*=0.307, *P*=0.010, respectively) and negatively correlated with myo-inositol (*r*=−0.431, *P*<0.001, *r*=−0.366, *P*=0.002, respectively). tNAA was significantly correlated with whole-brain and ACR FA in schizophrenia patients but not in controls (Table 3). Myo-inositol and FA were significantly correlated in the combined sample and both groups independently (Table 3 and Figure 2e).

### Relationship of age and FA after controlling for white matter metabolites

Controlling for each metabolite, it appears that the relationship between age and the frontal ROI FA remained unchanged or changed only slightly (Table 4), suggesting that the relationship between age and FA was not primarily controlled by the level of frontal white matter metabolites. The findings were similar using whole-brain FA.

### Omnibus test on contributions to FA

The above analysis identified several factors possibly associated with FA: age, diagnosis, white matter glutamate, NAA and myo-inositol. Therefore, we tested the overall model on how their effects may contribute to the frontal white matter ROI FA, in which left ACR FA=β0+β1 × Age+β2 × Glutamate+β3 × myo-inositol+β4 × tNAA+β5 × Diagnosis.

The overall model was significant (*R*^2^=37.6%, F_(5,63)_=7.60, *P*<0.001). Beside glutamate (*P*=0.36) and tNAA(*P*=0.056), all other factors significantly contributed to FA: age (*t*=−2.16, *P*=0.034), diagnosis (*t*=−2.86, *P*=0.006) and frontal white matter myo-inositol (*t*=−3.04, *P*=0.003), suggesting that schizophrenia, older age and higher white matter myo-inositol were each significant, independent predictors of reduced FA after controlling for all the other measures. The largest effect size was from myo-inositol. Critically, the negative impact of age and schizophrenia on FA cannot be fully accounted for by these metabolites in the same frontal white matter area. Variance inflation factors were <3 in each component in the model, indicating acceptable collinearity.

Exploring the same model using whole-brain FA, the findings were essentially the same. The overall model was significant (*R*^2^=49.9%, F_(5,63)_=12.54, *P*<0.001). Besides glutamate (*P*=0.65), all other factors significantly contributed to FA: age (*t*=−2.29, *P*=0.025), diagnosis (*t*=−2.88, *P*=0.005), frontal white matter myo-inositol (*t*=−4.12, *P*<0.001) and tNAA (*t*=2.91, *P*=0.005). Variance inflation factors were <3 in each component in the model. Finally, the correlation coefficients between age and whole-brain FA were significantly different between schizophrenia patients (*r*=−0.71) and controls (*r*=−0.36) (*z*=−2.07, *P*=0.038), replicating the previous report on a greater age-related FA decline in schizophrenia.^3^

### Clinical correlates

On smoking, univariate analyses of smoking vs diagnosis effects on FA and each of the metabolite showed a significant main effect of smoking on glutamate (*P*=0.011) but no significant smoking × diagnosis interaction (*P*=0.18). *Post hoc* test showed that smokers had higher frontal white matter glutamate than nonsmokers (F(1,68)=4.91, *P*=0.030). No other metabolite showed significant smoking or smoking × diagnosis interaction.

In the combined sample, body mass index (BMI) was significantly and positively correlated with white matter myo-inositol concentration (*r*=0.322, *P*=0.007) and this trend was evident in patients (*r*=0.341, *P*=0.052) and controls (*r*=0.410, *P*=0.014) analyzed independently. However, no other metabolite was significantly correlated with BMI (all *P*>0.05).

Correlation analyses with cognitive measures showed that processing speed was not significantly associated with any metabolites in controls, patients or the combined sample (all *P*>0.05). In schizophrenia patients, processing speed was significantly and positively associated with left frontal white matter FA (*r*=0.47, *P*=0.007). We found no significant correlation of these metabolites with working memory in either controls or patients (all *P*>0.05). Finally, none of the white matter metabolites or FA were significantly associated with Brief Psychiatric Rating Scale total scores or chlorpromazine dose equivalent in schizophrenia patients (all *P*>0.05).

## Discussion

We examined whether several white matter metabolites that change with healthy aging may contribute to lower white matter FA and greater FA decline with aging in schizophrenia. ACR FA value was significantly associated with schizophrenia, age and one white matter metabolite, that is, myo-inositol. Myo-inositol significantly and independently contributed to FA even after the other factors were controlled. However, we also found that the age-related decline in FA remained significant even after controlling for the metabolites we examined. This implicates the involvement of additional mechanisms in the greater age-related decline in FA in schizophrenia patients compared with controls (Figure 2a). Similarly, we found that reduced FA in schizophrenia patients remained significant after controlling for age and metabolites, again suggesting the involvement of additional mechanisms in the FA deficit in schizophrenia patients.

The finding that white matter myo-inositol demonstrated the strongest role in white matter FA is interesting. Previous studies of diseases with known white matter inflammation mechanisms have demonstrated increased myo-inositol levels as discussed in the Introduction section. Here, we found a significant, negative correlation between white matter myo-inositol and white matter FA. This finding is considered to be replicable because it is present in both normal controls and schizophrenia patients independently. We interpret this as evidence of an effect of inflammation on white matter microstructure, which is not specific to schizophrenia as the same trend was present in both patients and controls. We found that myo-inositol followed an expected positive trend with age (Figure 2d) as seen in other studies^36^ although it was not statistically significant. We also found an association between BMI and white matter myo-inositol, replicating previous studies.^68^ However, we did not find evidence that myo-inositol was abnormally elevated in schizophrenia, nor had a specific influence on white matter in schizophrenia. This is further supported by the omnibus multiple regression analysis, which showed that myo-inositol has a robust effect on FA; yet FA remained significantly and independently affected by diagnosis.

We previously reported that white matter NAA was significantly correlated with FA in normal controls using the data collected from a different cohort and a different scanner.^16^ This is replicated in the current sample. Our previous data demonstrated a stronger correlation in an older-age cohort, similar to the schizophrenia group in the current study. Accelerated aging has been hypothesized to occur in schizophrenia, and it is possible that the correlation between FA and NAA may resemble that observed in the older-age controls. However, we also found that the age-related decline in FA remained largely unchanged when controlling for white matter NAA levels (Table 4). This observation argues against white matter NAA as the primary cause of the steeper decline of FA in schizophrenia. Additional statistical tests confirmed this argument. Controlling for age, the relationship between frontal white matter FA and NAA essentially disappeared in controls (partial *r*=−0.07, *P*=0.68) and patients (partial *r*=0.02, *P*=0.92). Therefore, both white matter NAA and FA significantly declined with age, but NAA does not appear to explain the age-related decline in FA in schizophrenia patients.

There are several limitations in this study. First, this is a cross-sectional study and we have interpreted the FA and metabolite associations with age as reflecting an aging process. However, using cross-sectional data to infer longitudinal changes has significant limitations^69^ and confirmation of our findings requires an alternative, longitudinal design. Second, we cannot state with full certainty that the antipsychotic medications did not impact these results, though chlorpromazine equivalent dose was not associated with any white matter measures. However, chlorpromazine is likely an overly simplified approach to assess antipsychotic medication exposure. A lack of relationship with chlorpromazine should not be viewed as proof of the lack of impact of antipsychotic medication exposure on these imaging measures. Third, metabolites measured with MRS in this study have multiple functions; for example, myo-inositol may track effects of neuroinflammation, but also has a role in other glial and non-glial functions. Any direct reference to a particular signaling pathway should be viewed with caution. Fourth, it is possible that substance abuse or dependence in the past could have impacted our DTI or MRS data; we attempted to minimize this potential confound by excluding participants with more recent substance abuse or dependence (other than nicotine). Finally, our ability to interpret these data is limited by sampling MRS in a small white matter voxel. Our focus was on comparing DTI and MRS in the same frontal white matter voxel, assuming this might be more informative than comparing frontal white matter neurochemistry to whole-brain DTI; however, we found that the frontal DTI-MRS findings followed a similar pattern as the whole-brain DTI—frontal MRS findings. Without measuring whole-brain MRS, the reason for the similarity is difficult to interpret. Additional efforts will be needed to determine if there are meaningful differences in global vs local patterns of relationship between DTI and MRS parameters.

We believe this is the first study to combine MRS and DTI to study frontal white matter integrity as a function of age in schizophrenia. The overall purpose was to decipher the biological underpinnings of FA abnormalities including age-related decline in frontal white matter FA. We found some evidence suggesting that white matter neuroinflammation, as indexed by myo-inositol, may contribute to reduced FA in general. Greater decline in white matter FA and NAA were observed in schizophrenia (Figures 2a and c). However, the greater age-related frontal FA decline in schizophrenia cannot be explained by the neurochemistry assessed in this study. Further studies are warranted to determine the underlying mechanism driving the accelerated FA decline with age in schizophrenia.

## Figures and Tables

**Table 1:** Clinical and imaging information

	*SZ*	*NC*	*F or* χ^*2*^	P
*Clinical information*				
Age	39.3 (12.8)	39.1 (12.9)	0.00	0.97
Male (%)	74.4	66.7	0.53	0.61
Smoker (%)	53.8	22.2	7.89	0.009
BMI	29.7 (6.2)	26.3 (5.0)	6.50	0.013
Working memory	35.5 (14.0)	45.0 (12.6)	8.48	0.005
Processing speed	7.2 (3.0)	10.3 (2.4)	22.04	<0.0001
BPRS total mean	2.0 (0.47)	NA	NA	NA
				
*Imaging measures*				
Left frontal FA	0.45 (0.04)	0.47 (0.03)	6.20	0.015
Whole-brain FA	0.44 (0.04)	0.46 (0.02)	4.45	0.038
Left frontal glutamate	7.62 (1.28)	7.69 (1.30)	0.58	0.81
Left frontal NAA	9.13 (1.10)	9.11 (0.92)	0.00	0.95
Left frontal myo-inositol	5.05 (1.16)	5.30 (1.11)	0.84	0.36
Left frontal choline	2.12 (0.36)	2.25 (0.37)	2.25	0.138

Abbreviations: BMI, body mass index; BPRS, Brief Psychiatric Rating Scale; FA, fractional anisotropy of white matter; MRS, magnetic resonance spectroscopy; NA, not available; NC, normal controls; ROI, region of interest; SZ, patients with schizophrenia; NAA, *N*-acetylaspartate.

Except for gender and smoking status, all values represent means with standard deviation in parentheses. The four metabolites were measured from the frontal white matter. MRS-ROI represents the left frontal white matter voxel where MRS metabolite measurements were made.

**Table 2:** Correlation of age with white matter measures and in patients with schizophrenia (SZ) and normal controls (NC) separately

*White matter measures*	*SZ*		*NC*	
	r	P	r	P
Left frontal FA	−**0.55**	**0.001^a^**	−**0.41**	**0.012**
Whole-brain FA	−**0.71**	**<0.001^a^**	−**0.36**	**0.030**
Left frontal glutamate	−**0.44**	**0.010**	−**0.37**	**0.027**
Left frontal NAA	−**0.63**	**<0.001^a^**	−**0.36**	**0.032**
Left frontal myo-inositol	0.40	0.021	0.23	0.18
Left frontal choline	0.05	0.79	0.09	0.59

Abbreviations: FA, fractional anisotropy of white matter; NAA, *N*-acetylaspartate.

Nominally (*P*<0.05) significant correlations that were replicated across groups are in bold.

a Significant after Bonferroni correction for 12 comparisons (*P*<0.004).

**Table 3:** Correlation between white matter FA and white matter metabolites

*White matter MRS*	*Correlations with left ACR FA*				*Correlations with whole-brain FA*			
	*SZ*		*NC*		*SZ*		*NC*	
	r	P	r	P	r	P	r	P
Glutamate	0.25	0.16	0.07	0.71	0.46	0.006	0.00	0.98
NAA	0.48	0.004	0.08	0.65	0.64	<0.001^a^	0.07	0.70
Myo-inositol	**−0.45**	**0.008**	**−0.36**	**0.033**	**−0.45**	**0.008**	**−0.51**	**0.001**^a^
Choline	−0.11	0.79	−0.10	0.55	−0.13	0.47	−0.11	0.54

Abbreviations: ACR, anterior corona radiata; FA, fractional anisotropy of white matter; MRS, magnetic resonance spectroscopy; NC, normal controls; SZ, patients with schizophrenia; NAA, *N*-acetylaspartate and glutamate.

Nominally significant (*P*<0.05) correlations that were replicated across groups are in bold.

a Significant after Bonferroni correction for 16 comparisons (*P*<0.003).

**Table 4:** Partial correlations between age and FA (partial correlation coefficient *r* and *P*-values) after controlling for each of the measured white matter metabolites

*Controlled for*	*Left frontal FA*–*age partial correlation*				*Whole-brain FA*–*age partial correlation*			
	*SZ*		*NC*		*SZ*		*NC*	
	r	P	r	P	r	P	r	P
Glutamate	−0.51	0.002^a^	−0.42	0.01	−0.63	<0.001^a^	−0.39	0.02
NAA	−0.37	0.04	−0.41	0.01	−0.51	0.002^a^	−0.36	0.03
Myo-inositol	−0.46	0.007	−0.36	0.03	−0.65	<0.001^a^	−0.29	0.09
Choline	−0.55	0.001^a^	−0.41	0.02	−0.73	<0.001^a^	−0.36	0.04

Abbreviations: FA, fractional anisotropy of white matter; NC, normal controls; SZ, patients with schizophrenia; NAA, *N*-acetylaspartate.

Note that the correlation between age and FA was generally maintained, regardless of levels of metabolites.

a Significant after Bonferroni correction for 16 comparisons (*P*<0.003).
